# Effects of quercetin supplementation in aging mice on oocyte number, in vitro fertilization and embryo development outcomes

**DOI:** 10.5935/1518-0557.20260024

**Published:** 2026

**Authors:** Tanapak Wisetmongkolchai, Pannarai Somboonchai, Waraporn Piromlertamorn, Usanee Sanmee

**Affiliations:** 1 Division of Reproductive Medicine, Department of Obstetrics and Gynecology, Faculty of Medicine, Chiang Mai University, Thailand; 2 CMEx Fertility Center, Center of Medical Excellence, Chiang Mai University, Thailand

**Keywords:** quercetin, oocytes, aging mice, in vitro fertilization, embryo development

## Abstract

**Objective:**

To investigate the effects of quercetin supplementation in aging mice on the oocyte number and in vitro fertilization outcomes.

**Methods:**

Thirty-two to forty-week-old aging female mice were randomly assigned into two groups, with one receiving 0.2 mL water contains quercetin 30 mg/kg dissolve in DMSO orally via gavage for 21 consecutive days (n=38). The control group was given 0.2 mL of water containing DMSO without quercetin (n=37). In vitro fertilization was performed after exogenous ovarian stimulation. The outcome was oocyte number and quality, as indicated by oocyte fragmentation rate, fertilization rate, blastocyst formation rate, and blastocyst cell number.

**Results:**

There was no difference in the number of retrieved oocytes per mouse (17.4±2.6 and 16.2±1.7, *p*=0.694), oocyte fragmentation rate (30.8% and 26.7%, *p*=0.108), fertilization rate (43.5% and 43.1%, *p*=0.877) and blastocyst formation rate (32.6% and 33.9%, *p*=0.635) in the quercetin group compared to the control group. Likewise, the mean numbers of cells in the ICM, TE, and total cell number in blastocysts from both groups were not different.

**Conclusions:**

Dietary quercetin supplementation did not improve either the quantity or quality of oocytes. Thus, quercetin supplementation did not improve IVF outcomes in aged mice. However, future studies should incorporate oxidative stress biomarkers to improve mechanistic understanding.

## INTRODUCTION

Currently, the global fertility rate continues to decline, and many countries are facing a change in their population structure. As the elderly population increases, the young and working-age population decreases. Women tend to delay having children (Hong *et al*., 2022; Aitken, 2022), which increases the likelihood of infertility due to declining oocyte quantity and quality. Aging women are at increased risk of chromosomal abnormalities and miscarriage, which cannot be eliminated by assisted reproductive technology (ART) treatment. Ovarian aging is a natural and inevitable process; however, its exact mechanisms remain incompletely understood. A widely accepted hypothesis is that the free radical theory of aging induces oxidative stress, resulting from an imbalance between reactive oxygen species (ROS) and antioxidants. The impairment of cellular repair mechanisms diminishes oocyte quality and quantity (Wang *et al*., 2021; Baghel *et al*., 2012).

In response to the challenges of delaying aging and improving fertility outcomes, numerous antioxidants such as CoQ10, vitamin C, vitamin E, astaxanthin, apocynin, and melatonin have been widely used (Shang *et al*., 2024; Abdollahifar *et al*., 2019; Tarín *et al*., 2002; Okamoto *et al*., 2022; Timóteo-Ferreira *et al*., 2019; Liu *et al*., 2012). In Thailand, kaffir lime juice, which is rich in quercetin, is commonly used as a dietary supplement by infertile couples despite its unproven efficacy and safety (Vitagliano *et al*., 2021; Štochmaľová *et al*., 2013). Quercetin is a potent flavonoid reported to scavenge ROS, inhibit lipid peroxidation, and modulate antioxidant enzyme expression (Baghel *et al*., 2012; Wang *et al*., 2018; Zhu *et al*., 2023). Nevertheless, evidence regarding its benefits on the quantity and quality of oocytes remains controversial. Our previous study (Chantrasiri *et al*., 2025) found that dietary quercetin supplementation in reproductive-aged mice, 6-12 weeks old, did not increase the number of retrieved oocytes nor enhance the oocyte quality, and did not increase fertilization rate and blastocyst formation rate. Moreover, blastocyst quality was even lower in the quercetin-supplemented group, which raises concerns about the widespread use of dietary quercetin supplements in infertile females. However, Naseer *et al*. (2017) and Jahan *et al*. (2018) demonstrated the contrasting results, showing improvements in follicular development and oocyte quality with quercetin supplementation. This could perhaps be explained by the fact that they induced oxidative stress conditions by exposing heat stress (Naseer *et al*., 2017) or inducing polycystic ovarian syndrome (PCOS) (Jahan *et al*., 2018), while we use the normal condition without stress on animals (Chantrasiri *et al*., 2025).

There is robust evidence that aging is associated with an imbalance in redox state due to the decrease in activity of the naturally occurring antioxidant enzymes, causing oxidative stress (Wang *et al*., 2021). Therefore, we decided to investigate the effects of dietary quercetin in aging mice on the oocyte number and in vitro fertilization (IVF) outcomes. We hypothesized that quercetin might enhance oocyte outcomes and embryo quality by counteracting oxidative stress, a key contributor to ovarian aging.

## MATERIALS AND METHODS

The experiments were approved by the Institutional Animal Care and Use Committee of Chiang Mai University (Protocol number 33/2567). Investigators are certified by the Institute of Animals for Scientific Purpose Development (IAD), and the National Research Council of Thailand (NRCT).

### Animals

The International Cancer Research (ICR) mice were sourced from the National Animal Institute, Mahidol University, Bangkok, Thailand. Female mice, aged 32-40 weeks, with a body weight of 50±10 g, and male mice, aged 10-12 weeks, were used in the study. All mice were housed in the Animal Husbandry Unit of the Faculty of Medicine, Chiang Mai University, under controlled conditions, room temperature at 25±2°C, humidity level of 60-70%, and a 12-hour light/dark cycle. Food pellets and tap water were provided ad libitum. All experimental procedures were performed according to national and international ethical guidelines for animal care.

### Experimental design

Quercetin (Q4951, Sigma-Aldrich, St. Louis, USA) was dissolved in dimethyl sulfoxide (DMSO; Sigma-Aldrich, St. Louis, MO, USA). Based on our previous study (Chantrasiri *et al*., 2025), a negative effect of dietary quercetin on embryo quality without any benefit on the ovary of young mice, even with quercetin doses similar to those previously reported to be beneficial on ovarian follicles (Naseer *et al*., 2017; Jahan *et al*., 2018). Therefore, we decided to use the same dosage of 30 mg/kg of quercetin and the same experimental design in this study to see the effect of dietary quercetin on the aging mice.

Thirty-twoto forty-week-old female mice were randomly assigned to two groups: the control group (receiving DMSO in distilled water) and the quercetin group (receiving quercetin dissolved in DMSO and diluted with distilled water).

Mice received 0.2 mL of water with or without quercetin supplementation daily via oral gavage for 21 consecutive days. To ensure accurate results, the experiment was repeated four times and the results from all four runs were combined for the final analysis. A supplemented solution was prepared weekly, maintaining a quercetin dose of 30 mg/kg ensuring the same DMSO concentration between the two groups.

### Collection of cumulus - oocyte complexes (COCs)

On the day following 21 days of supplementation, ovarian stimulation was induced by administering 10 IU of Pregnant Mare Serum Gonadotropin (PMSG; Sigma, St. Louis, MO, USA) via intraperitoneal (IP) injection. After 48 hours, 10 IU of human chorionic gonadotropin (Pregnyl, Organon, Oss, The Netherlands) was administered intraperitoneally, followed by euthanasia 16 hours later using the cervical dislocation method. The peritoneal cavity was exposed, and both oviducts were aseptically removed and placed in Earle’s Balanced Salt Solution (EBSS; Biological Industries, Kibbutz Beit Haemek, Israel) containing 0.5% bovine serum albumin (BSA; Sigma, St. Louis, MO, USA). The oviducts were carefully incised with a 30-gauge needle under a microscope to release the COCs, which were subsequently transferred to fertilization medium (G-IVF, Cook) for insemination. Mice that died during supplementation or failed to respond to superovulation injections were excluded from the study.

### In vitro fertilization and embryo culture

Sperm were collected from the cauda epididymis of ten to twelve-week-old male mice following euthanasia by cervical dislocation and placed in a 5 mL cell culture tube containing 1 mL of fertilization medium. The tube was then incubated in a CO₂ incubator for 30 minutes to allow sperm to migrate to the surface of the medium (swim-up technique). The upper layer containing motile sperm was carefully collected and used for fertilization with COCs at a concentration of 300,000 motile sperm/mL. Control and quercetin groups used sperm from the same male mouse.

After two hours, MII oocytes were washed to remove excess sperm and transferred to cleavage medium (G1-plus; Vitrolife, Sydney, Australia) under mineral oil (Irvine Scientific). The culture was maintained at 37°C, 6% CO₂, 5% O₂, and 89% N₂. After 72 hours post-fertilization, embryos were transferred to blastocyst medium (G2-plus; Vitrolife, Sydney, Australia). The development of embryos was recorded every 24 hours until 120 hours post-insemination. Blastocysts were classified based on Gardner’s morphological criteria: early, partial, full, expanding, hatching, and hatched blastocysts (Gardner *et al*., 2000).

### Differential staining of the inner cell mass (ICM) and trophectoderm cell (TE)

All full, expanding, hatching, and hatched blastocysts at 120 hours post-insemination underwent differential staining. Blastocysts with an intact zona pellucida were first immersed in 0.5% pronase (Sigma P8811) for ten minutes to remove the zona. The zona-free blastocysts were then rinsed three times in sodium-, calcium-, and magnesium-free phosphate-buffered solution (PBS; Gibco, USA) before being incubated in rabbit anti-mouse antibody (Sigma M5774; 1:1 dilution) at 37°C for 30 minutes. After an additional PBS wash, the blastocysts were transferred into a solution containing guinea pig complement serum (Sigma S1639), 20 µg/mL propidium iodide (Sigma P4170), and 10 µg/mL Hoechst dye, and incubated at 37°C for 15 minutes until the trophectoderm (TE) and inner cell mass (ICM) became swollen. In this process, propidium iodide stained the TE nuclei red, while Hoechst stained both the ICM and TE nuclei blue (Van Soom *et al*., 2001). Finally, each blastocyst was washed, placed on a glass slide, and air-dried before being covered with a coverslip and mounted with glycerol.

To count the TE and ICM cell numbers, a Nikon Eclipse 80i fluorescent microscope connected to a Nikon DS-Ri2 digital camera equipped with a barrier filter of 590 nm and an excitation filter of 515-560 nm was used to photograph and detect the red-stained and blue-stained nuclei, respectively. The images were then merged and analyzed using the NIS-Elements program version 5.10.01 (Laboratory Imaging, Yokohama, Japan).

### Statistical analysis

Statistical analysis was performed using SPSS version 28.0.1.1. The number of oocytes, oocyte fragmentation rate, fertilization rate, and blastocyst formation rate between the two groups were compared using the chi-square test or Fisher’s exact test as appropriate. For normally distributed data, an independent t-test was used to compare the mean numbers of ICM, TE, total cells, and the ICM:TE ratio; otherwise, the Mann-Whitney U test was applied. A *p*-value of < 0.05 was considered statistically significant.

## RESULTS

A total of 80 mice were initially included in the study, with 40 mice assigned to each group. During treatment, two mice in the control group and one mouse in the quercetin group died, and one mouse from each group failed to respond to the superovulation injection. Finally, a total of 75 mice were included in the analysis, 37 mice in the control group and 38 mice in the quercetin group. There were no significant differences observed for any parameter. No significant differences were observed in the number of recovered oocytes per mouse (17.4±2.6 *vs*. 16.2±1.7, *p*=0.694), oocyte fragmentation rate (30.8% *vs*. 26.7%, *p*=0.108), fertilization rate (43.5% *vs*. 43.1%, *p*=0.877), and blastocyst formation rate (32.6% *vs*. 33.9%, *p*=0.635) in the quercetin group compared to the control group (Table 1).

**Table 1:** Effects of dietary quercetin on oocyte number, oocyte fragmentation rate, fertilization rate, and blastocyst formation rate

	Control	Quercetin	*p*-value
No. of mice	37	38	-
Total collected oocytes	599	662	-
No. of oocyte recovered/mouse^^*^^	16.2±1.7	17.4±2.6	0.694
Oocyte fragmentation rate	160 (26.7)	204 (30.8)	0.108
Fertilization rate	258 (43.1)	288 (43.5)	0.877
Blastocyst rate	203 (33.9)	216 (32.6)	0.635

Values are presented as numbers (%), Chi-square test, unless otherwise stated

* mean±standard deviation, t-test

Blastocyst development after 120 hours post-insemination is shown in Table 2. The proportion of early, partial, full, expanding, hatching, and hatched blastocysts was comparable in the quercetin and the control group.

**Table 2:** Mouse embryo development after 120 hours of culture

	Control	Quercetin	*p*-value
No. of blastocyst	203	216	-
EB	14 (6.9)	20 (9.3)	0.377
B	25 (12.3)	17 (7.9)	0.131
FB	27 (13.3)	30 (13.9)	0.861
ExB	28 (13.8)	24 (11.1)	0.406
HB	95 (46.8)	104 (48.1)	0.782
HtB	14 (6.9)	21 (9.7)	0.297

Values are presented as numbers (%), Chi-square test

EB, early blastocyst; B, partial blastocyst; FB, full blastocyst; ExB, expanding blastocyst; HB, hatching blastocyst; HtB, hatched blastocyst

There was no significant difference in the mean numbers of cells in the ICM (25.3±12.5 *vs*. 27.0±12.8, *p*=0.375), TE (88.0±29.4 *vs*. 87.9±27.3, *p*=0.986), and total cell number (113.3±37.3 *vs*. 114.9±35.5, *p*=0.767) in blastocysts from the quercetin and the control group (Table 3). Likewise, the ICM to TE ratio did not differ between groups (0.29±0.12 *vs*. 0.31±0.13, *p*=0.295).

**Table 3:** Numbers of cells in the ICM, TE, total cell number, and the ICM to TE ratio

	Control	Quercetin	*p*-value
ICM	27.0±12.8	25.3±12.5	0.375
TE	87.9±27.3	88.0±29.4	0.986
Total cell	114.9±35.5	113.3±37.3	0.767
ICM/TE ratio	0.31±0.13	0.29±0.12	0.295

Values are presented as mean±standard deviation, t-test

ICM, inner cell mass; TE, trophectoderm cell

## DISCUSSION

Advanced maternal age is associated with reduced fertility due to decreased oocyte number and quality. Therefore, most women who are undergoing infertility treatment are asking for fertility supplements with the anticipation of increasing their chances of getting pregnant. A significant number of supplements for fertility health are available on the market from several companies. Undeniably, antioxidants are the most popular product. We are interested in dietary quercetin, although it is not a commonly used or frequently studied antioxidant like vitamin C, vitamin E, or coenzyme Q10. It is an active ingredient in kaffir lime juice which is widely drunk among infertile women in our country nowadays. Because of the controversial effect of quercetin on ovarian function and the lack of IVF outcomes, we studied its effects with unexpected inverse associations of dietary quercetin intake with embryo quality in young mice (Chantrasiri *et al*., 2025). The study showed that dietary quercetin did not increase the number of retrieved oocytes and did not enhance the oocyte quality. Moreover, the resulting blastocyst from mice supplemented with dietary quercetin is of lower quality as measured by the proportion of hatching and hatched blastocyst and the blastocyst cell number, leading the concerns about its general use in infertile women and raising the question about its effect on the aging population. Our previous study supports the hypothesis that the balance between ROS generation and elimination is essential for optimal ovarian function (Yuan *et al*., 2012). Excessive exogeneous antioxidants could lead to the disruption of ovarian cellular redox balance. Therefore, antioxidants are likely to benefit conditions at risk for oxidative stress from either depletion of antioxidants or accumulation of ROS. An older age is associated with increased ROS and decreased antioxidant levels in the oocyte and ovarian environment (Tatone *et al*., 2006). A recent meta-analysis (Shang *et al*., 2024) concluded that antioxidant consumption for women with ovarian aging significantly increased the number of oocytes retrieved, high-quality embryos, and a higher clinical pregnancy rate than non-supplemented. However, the antioxidants used in this review do not include quercetin.

Our PubMed search, using the terms quercetin and ovarian aging, found only two publications on this subject, and both studied animals (Wang *et al*., 2018; Beazley & Nurminskaya, 2016). They investigated the effect of quercetin on the change in hormone levels, antioxidant markers, and natural fertility, no study has been carried out on IVF outcomes after quercetin supplementation. Wang *et al*. (2018) showed that quercetin at low-dose (12.5 mg/kg), middle-dose (25 mg/kg) and high-dose (50 mg/kg) do not affect the levels of estrogen, progesterone, as well as follicle stimulating hormone (FSH) and luteinizing hormone (LH) after 90 days supplemented in the menopausal rat. Although the increase in expression of oxidative stress-related genes such as superoxide dismutase-1 (SOD-1), catalase (CAT) and glutathione synthetase (GSS) was detected but the serum levels of total antioxidant capacity (T-AOC), superoxide dismutase (SOD), glutathione (GSH), glutathione peroxidase (GSH-PX) and glutathione-S-transferase (GST) were not significant changes. This study indicated that dietary quercetin supplementation, even at low-doses, was sufficient to upregulate the expression of oxidative stress-related genes in the ovary. However, the effect on ovarian function is difficult to conclude because the study was conducted in the menopausal rat whose ovary had naturally stopped functioning. Beazley & Nurminskaya (2016) studied the effect of dietary quercetin on the natural conception of mice using a dose of 5 mg/kg/day for nine months, starting at four weeks old. When bred at 8-11 months defined as a near reproductive cessation age in mice, the number of mice born per litter was significantly lower in quercetin-treated mice compared to untreated mice (3.0±0.1 and 5.2±0.2).

The results of the present study on the IVF outcomes demonstrated that dietary quercetin supplementation in aging mice at 8-10 months of age did not increase the number of retrieved oocytes nor enhance the oocyte quality. The fertilization rate, blastocyst formation rate, and blastocyst quality, as indicated by blastocyst morphology and cell numbers, were similar to those in the non-supplemented group. Our results were not consistent with previous studies that reported improvement in ovarian function and reproductive outcome during aging with other dietary antioxidant supplementation besides quercetin (Abdollahifar *et al*., 2019; Tarín *et al*., 2002; Okamoto *et al*., 2022; Timóteo-Ferreira *et al*., 2019; Liu *et al*., 2012; He *et al*., 2023). This may be due to, firstly, the dose and duration of dietary quercetin supplementation in our study might not be optimal for the aging ovary. The dosage of 30 mg/kg and the 21 days duration of supplement extrapolated from studies that reported the advantages of dietary quercetin on the ovary, but it was a studies on different species, rabbits (Naseer *et al*., 2017) and rats (Jahan *et al*., 2018), and in the oxidative stress induced condition, not the aging ovary. It is not clear what dose of quercetin and administration protocols are optimal for an ovarian aging population. Therefore, one limitation of this study is the lack of oxidative stress biomarkers which are the important tools to assess the redox status of the aging mice used in the study and the effects of the antioxidant quercetin. Future studies should incorporate oxidative markers to improve mechanistic understanding. Secondly, quercetin may not be the antioxidant of choice for ovaries. Ovarian aging may be ameliorated with specific antioxidant molecules. Our literature review shows that vitamin C supplements increase the number of primordial, primary, secondary, and antral follicles in the ovary and the number of retrieved oocytes in aging mice (Abdollahifar *et al*., 2019; Tarín *et al*., 2002). The study on vitamin E supplements showed an increase in number of ovarian oocytes and the total number of oocytes retrieved from the oviduct and ovary of aging mice (Tarín *et al*., 2002). Astaxanthin supplements increase follicular number in the ovary of aged laying hens (He *et al*., 2023). Apocynin supplements increase follicular number in the ovary of aged mice (Timóteo-Ferreira *et al*., 2019). N-acetyl cysteine supplements increased the litter size of aging mice and also increased the quality of oocytes as indicated by fertilization rate and embryo development (Liu *et al*., 2012). To date, to our knowledge, no clinical study has shown the advantages of dietary quercetin on oocyte number and quality, and IVF outcomes in ovarian aging. Lastly, the mechanism underlying ovarian aging is complex and remains incompletely understood. Multiple contributing factors cause ovarian aging, not only oxidative stress. Certainly, taking a single antioxidant supplementation could not overcome the multifactorial nature of ovarian aging.

Although the present study did not show any benefit of quercetin on the ovary, it also did not show any negative effects, which was in contrast to the finding by Beazley & Nurminskaya (2016). This may be explained by long term dietary quercetin supplements starting from young mice, through reproductive age, and into old age mice. As we know, low levels of ROS are essential for cell function, cell growth, and the controlled cell death pathway (Mauchart *et al*., 2023). Over exogeneous antioxidant supplements disrupted this redox balance. Based on our present findings and a previous study on the effect of quercetin on the ovary (Chantrasiri *et al*., 2025; Beazley & Nurminskaya, 2016). We emphasized that quercetin did not improve the ovarian quantity and quality in aging ovaries, and it has an unwanted effect on female fertility if used in the long-term or at a young age without a particular condition that requires antioxidant treatment. These findings raise concerns about the widespread, yet unverified, use of kaffir lime juice supplements by Thai infertile couples and highlight the need for more research to relate our findings.

In conclusion, our study indicates that dietary quercetin supplementation did not show any beneficial effect on the aging ovary in both quantity and quality, Therefore, it does not enhance IVF outcomes on aged oocytes.
